# Associations between Urban Green Spaces and Health are Dependent on the Analytical Scale and How Urban Green Spaces are Measured

**DOI:** 10.3390/ijerph16040578

**Published:** 2019-02-16

**Authors:** Liqing Zhang, Puay Yok Tan

**Affiliations:** Department of Architecture, School of Design and Environment, National University of Singapore, Singapore 117566, Singapore; puay.yok.tan@nus.edu.sg

**Keywords:** urban green spaces, self-reported mental health, measurement of green spaces, spatial scale, Singapore

## Abstract

Although the benefits from exposure to urban green spaces (UGS) are increasingly reported, there are important knowledge gaps in the nature of UGS-health relationships. One such unknown area is the dependence of UGS-health associations on the types of UGS studied, the way they are quantified, and the spatial scale used in the analysis. These knowledge gaps have important ramifications on our ability to develop generalizations to promote implementation and facilitate comparative studies across different socio-cultural and socio-economic contexts. We conducted a study in Singapore to examine the dependence of UGS-health associations on the metrics for quantifying UGS (vegetation cover, canopy cover and park area) in different types of buffer area (circular, nested and network) at different spatial scales. A population-based household survey (*n* = 1000) was used to collect information on self-reported health and perception and usage pattern of UGS. The results showed that although all three UGS metrics were positively related to mental health at certain scales, overall, canopy cover showed the strongest associations with mental health at most scales. There also appears to be minimum and maximum threshold levels of spatial scale at which UGS and health have significant associations, with the strongest associations consistently shown between 400 m to 1600 m in different buffer types. We discuss the significance of these results for UGS-health studies and applications in UGS planning for improved health of urban dwellers.

## 1. Introduction

The health benefits of urban green spaces (UGS) have been widely documented in different disciplines such as public health, environmental psychology, and urban ecology, supported by evidence gathered from an increasing number of empirical studies over the past two to three decades. However, as highlighted by a recent review [1], there are important inconsistencies between studies, one of which is that the magnitude of health benefits differ in different studies, even when the same methods have been used to assess health responses from green space exposure. Why do some studies show significant health benefits while others only show marginal or an absence of positive health responses? Several reasons have been suggested: different types of UGS emphasized in the studies, different ways in which UGS was measured, and different spatial scales used in different studies [1]. There are also other reasons, such as the role of socio-cultural and socio-economic context, the influence of socio-demographic variables such as age, income, education, or urban morphology, such as differences between dense, compact and high-rise environment compared to sprawling urban areas. 

We suggest that as we continue to build up the knowledge base, it is necessary to first address methodological issues related to definition and measurements of UGS and analytical scale so that different studies can be made more comparable. For instance, UGS has a broad definition, and can refer to a plethora of vegetation types or green open spaces in the urban environment, such as nature areas, woodlands, parks, roadside vegetation, roof gardens, front and back yards, etc. Studies therefore need to explicitly state types of UGS studied in order for studies to be comparable [2]. Even when the same definition of UGS was used, the ways UGS were measured could well differ, depending on data availability or emphasis of the research. A common measurement method is to use satellite images or land cover map to distinguish different green space types and then calculate the presence, the number, the area, or the normalized difference vegetation index (NDVI) of green spaces linked to respondents’ residential address [3,4,5,6]. Studies have also used the number of trees (e.g., [7]) or tree canopy (e.g., [8]) to measure green space quantity. Some more complicated measures, such as vegetation diversity (e.g., [9]) and tree species composition (e.g., [10]), have also been used. There have also been some attempts to test the associations of different types of UGS with health [11,12].

Another important factor is that the spatial extent considered in the associations between UGS and health varies remarkably between studies. Most studies set a buffer size to prescribe the range of green spaces [6]. The setting of buffer size is normally based on the assumption that this range can represent the neighborhood environment where people spend their time sitting, walking, playing, or interacting with neighbors (e.g., [13,14,15,16,17,18]). The specific buffer size varies from study to study, such as 300 m [18], 500 m [17], 800 m [19], 1 km [15], 3 km [20], 5 km [13], depending on the definition of an individual’s neighborhood, specific health outcome, age group, and presumed mechanisms [6]. In addition, studies also use the administrative boundary to limit the study area, such as neighborhood scale, census unit scale, and city scale [1]. Results from different studies indicate that the scale of analysis influences the outcome of results. For instance, there are generally no or poor relationships between green space and health in studies conducted at larger levels, such as city scale [21,22], whereas at smaller spatial scales, results are mixed [4,23,24]. The effects of spatial scale on the measurement of a variable is a well-known problem in geographical studies known as the uncertain geographic context problem (UGCoP) [6,25]. Although there are studies comparing UGS at several buffers (e.g., [11,26,27,28,29]), how spatial scale of analysis influence conclusions derived from UGS-health studies is still not fully understood.

This paper reports on a study conducted in Singapore to assess the effects of the two uncertainties highlighted above: measurements of UGS and scale of analysis. The amount of vegetation has been widely measured in this field and found to be associated with improved health [12,15,30,31]. In addition, previous studies highlighted that tree canopy deserves particular consideration for its importance in public health [12,32,33], and in recent years, Singapore has seen a growing interest in urban parks and their potential for promoting health [34,35,36]. Thus, in this study, vegetation cover, canopy cover, and park area were selected as three different metrics to quantify UGS to examine their influence on UGS-health relationships.

The selection of scale of analysis was based on the results from the Park Usage and Satisfaction Survey conducted by National Parks Board (NParks) (the national government agency responsible for green space planning and management in Singapore) in 2009. This national-level survey reported that five minutes is the maximum travel time that frequent users are willing to spend to visit a neighborhood park [37]. The Euclidean distance for five-minute walking is generally accepted as 400 m. As suggested by a recent review on the scale issue [38], nested buffers are useful for examining the scale effects. Therefore, 400 m, 400–800 m, 800–1200 m, 1200–1600 m, 1600–2000 m, 2000–2400 m, 2400–2800 m were used as nested buffers to represent the space range with walking distance of 5 mins, 5–10 mins, 10–15 mins, 15–20 mins, 20–25 mins, 25–30 mins, and 30–35 mins, respectively. In contrast to a recent literature which calculated the weighted UGS exposure by assigning different weight for each nested buffer [39], the purpose of introducing nested buffers in this study is to distinguish the association of nearby greenery and distant greenery with mental health. To obtain a better understanding of the scale dependence of UGS-health relationships, a range of circular buffers with the radius of 50 m, 100 m, 200 m, 300 m, 400 m, 500 m, 600 m, 700 m, 800 m, 1000 m, 1200 m, 1600 m, 2000 m, 2400 m, and 2800 m were applied to measure UGS quantity. It is still uncertain whether visual contact with UGS is enough to bring mental health benefits or if direct use of UGS is needed for detectable health benefits. Direct use of UGS would still be a possible mechanism underlying the relationship between UGS quantity and mental health. As it has been suggested that network buffers based on road networks can better examine the usable greenness [38], in this study, eight network buffers with the distance of 400 m, 800 m, 1000 m, 1200 m, 1600 m, 2000 m, 2400 m, 2800 m were generated to compare the effects of circular buffers and network buffers.

The overall aim of this study is to compare the associations between three UGS metrics: vegetation cover, canopy cover and park area at multiple spatial scales, and self-reported mental health. The research questions and corresponding hypotheses are as follows. 

Research question 1: What are the associations between the quantity of each UGS metric and self-reported mental health?

**Hypothesis** **1.** *Significant and positive but weak associations between the amount of each UGS metric and mental health can be found at certain scales. As trees have large stature and biomass, they will have larger impact than grass on visual greenness and the environmental improvement; thus, it can be hypothesized that canopy cover has higher association with mental health than vegetation cover alone.*

Research question 2: How does the strength of associations between UGS quantity and self-reported mental health change with the scale used for analysis?

**Hypothesis** **2.** *Analytical scale has variable influence on the strength of association between different UGS metrics and mental health. At a very small scale, such as within several meters around a building, the amount of UGS may be too minimal to exert a biophysical (such as mitigation of heat or air pollution), or socio-cultural (such as providing recreational space) effect. Conversely, at very large spatial extent, very distant UGS is out of people’s daily commute range. Moreover, a very large scale would produce substantial overlap of greenery, and this may lead to a small variance of UGS quantity, which can hinder the observation of significant results. It is thus hypothesized that the association of UGS with health are more strongly observed at spatial scales in between the minimum and maximum threshold level. Given that more frequent green space usage is habitual, nearby green spaces will tend to be more often used on a routine basis. Proximate green spaces will thus tend to have a stronger association with health than distant green spaces.*

## 2. Materials and Methods

### 2.1. Study Background and Household Survey

This study is part of a larger project called “The Assessment of Dose-response Relationships between Urban Green Spaces and Self-reported Health”. According to the dose-response conceptual framework developed earlier [1], a household questionnaire survey based on a national representative sample of 1000 persons in Singapore was conducted to collect respondents’ self-reported health, perception of UGS, and use of UGS, as well as potential moderators and mediators. The ethical aspects of all the procedures have been reviewed and approved by Institutional Review Board of National University of Singapore (approval number A-16-383E). All methods in this study strictly followed the relevant guidelines and regulations.

The sampling frame was obtained from Singapore Department of Statistics (DOS) to ensure samples are representative of Singapore residents. The DOS main sampling methodology is based on a two-stage cluster design. The primary sampling units for the first stage of sample selection are sampling divisions which are the geographical units called “planning areas” (https://data.gov.sg/dataset/master-plan-2014-planning-area-boundary-web) in national land use planning in Urban Redevelopment Authority (URA). In the second stage of sampling, the sampling units are “dwelling units”, which are individual residential households. The sample selected based on this design had broad housing type distribution close to the population and would also capture various socio-economic characteristics of the different general population groups in Singapore. The corresponding block-level addresses of all dwelling units were also purchased from DOS for the spatial assessment of UGS around each dwelling unit. No identifiable personal information was linked to individual subjects. All personal identifiers were discarded at the end of the research study. The target on the proportion of gender, ethnicity and broad housing type distribution for residential dwellings were obtained from Singstat Table Builder (http://www.tablebuilder.singstat.gov.sg/publicfacing/mainMenu.action) in June 2016. 

A professional survey company was engaged to conduct the survey. Trained interviewers visited all household addresses provided for at least three attempts and selected an eligible respondent using the “Last Birthday” method, which involved selecting the people in the household with the most recent birthday. The criteria used for the initial screening was Singapore citizenship or Permanent Resident status, aged 21 years old and above, and a minimum one-year stay in the selected household address. One-year was selected as the minimum length of stay to yield evident health benefits from UGS following a previous study [40]. The household survey was conducted between June 2017 and December 2017, with a total sample size of 1000. The differences between the sample’s socio-demographic structure and the targeted ones were all less than 5%. The sample was thus comparable with the population profile of Singapore residents (Table 1).

In the household survey, mental health was measured using the 12-item General Health Questionnaire (GHQ-12), which has been widely used in UGS and health studies [41,42,43,44,45,46] and validated as an effective measure for Singapore population [47,48]. The GHQ-12 assesses the ability to conduct normal functions and the appearance of psychological distress by asking both positive (e.g., feeling useful, able to concentrate, etc.) and negative (e.g., lost sleep, feeling worthless, etc.) states. Answers indicating positive state was coded as 0 and answers indicating negative state was coded as 1. For the 12 items in mental health measure, the samples obtained a high degree of internal consistency (Cronbach’s alpha = 0.87). Following the scoring method used in previous studies [48,49], the total score for all questions were added, and a cut-off total score of 3 was used to determine good mental health (sum score: 0–2) and poor mental health (sum score: 3–12). Good mental health was then coded as 1 while poor mental health was coded as 0. 

The household survey questionnaire also collected other individual level data to screen confounding factors. Socio-demographic factors were recorded using standardized questions, covering gender, age, ethnicity, education level, marital level, employment status, residential status, housing ownership, housing type, individual and household income, and number of children in the house. The length of stay at the residence was also obtained, which has been suggested to be a proxy of neighborhood self-selection [50]. The respondents’ level of cigarette smoking and alcohol drinking behavior were also collected. 

Another behavioral factor is the level of physical activities. Most of the studies only measured the whole level of physical activities using one single indicator [20,43,51]. To be more precise, this study measured both indoor physical activities and green physical activities conducted in UGS. The level of indoor physical activities was measured using Godin-Shephard Leisure-Time Physical Activity Questionnaire [52] through this question: “during a typical 7-Day period (a week), how many times on the average do you do indoor strenuous, moderate, and mild physical activities for more than 15 minutes”. The frequencies of “strenuous”, “moderate”, and “mild” activities were multiplied by 9, 5, and 3 respectively. The weekly indoor physical activities score was then produced by summing up these three products. Similarly, the level of mild, moderate, and strenuous physical activities conducted in green spaces were also asked.

In addition, respondents’ Body Mass Index (BMI), level of health problems, sedentary behavior, time spent in travel from and to any places were collected as potential confounding factors. In addition, community involvement was measured to represent the social cohesion level by asking respondents’ agreement for the statement “if there is a need for community activities (e.g., cleaning public space, maintaining greenspace, etc.), people in my neighborhood would likely be involved” using a five-point scale (“disagree strongly”, “disagree”, “neutral”, “agree”, “agree strongly”) [53].

### 2.2. Assessment of UGS Quantity at Different Scales

**Figure 1:**
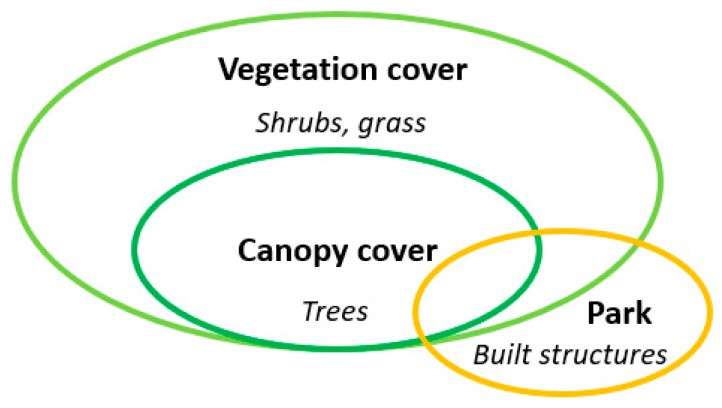
Venn diagram of three metrics of urban green spaces (UGS).

In this study, the amount of UGS were measured using three different metrics: vegetation cover, canopy cover, and park area. These metrics capture different aspects of UGS. As shown in Figure 1, canopy cover is a subset of vegetation cover, parks overlap with both vegetation cover and canopy cover. The island-wide vegetation cover and canopy cover was obtained from an earlier study [54]. These spatial data were derived from WorldView-2 satellite images with 2-m resolution for Singapore acquired in 2015. The polygons of park area, including both parks and nature reserves, were obtained from the Singapore Land Authority (SLA).

**Figure 2:**
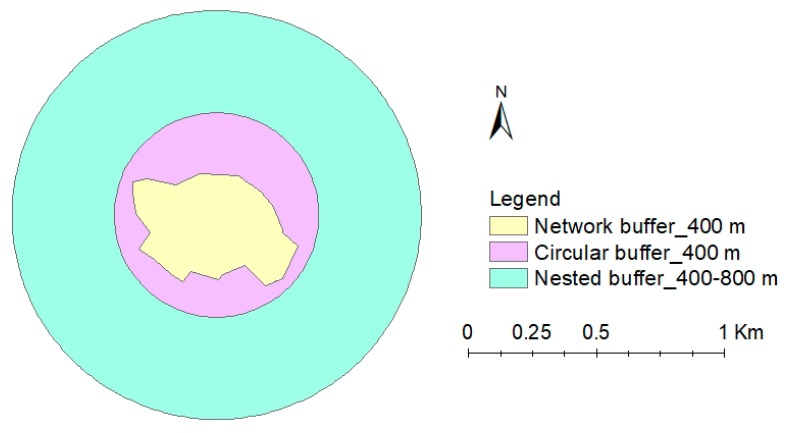
An example map of three types of buffers.

The postcodes of participants’ residence collected from the household survey were converted to coordinates through GPS Visualizer online (http://www.gpsvisualizer.com/convert_input). Using “Add XY data” in ArcGIS 10.3.1, the layer of corresponding points for all the coordinates was generated. Based on this point layer, three different types of spatial scales were created in ArcGIS for further spatial analysis (see the example in Figure 2). The first type of scale is the circular buffer around the residence with a specified straight-line Euclidean distance as its radius. By setting the buffer radii as 50 m, 100 m, 200 m, 300 m, 400 m, 500 m, 600 m, 700 m, 800 m, 1000 m, 1200 m, 1600 m, 2000 m, 2400 m, and 2800 m, the corresponding circular buffers were generated. The second type of spatial scale is the nested buffer with annular shape. Seven different nested buffers (400 m, 400–800 m, 800–1200 m, 1200–1600 m, 1600–2000 m, 2000–2400 m, 2400–2800 m) surrounding each respondent’s residential address were generated. The third type of scale is road network buffer with a specified distance representing the maximum distance that can be reached along the road network. The polyline data of road network was obtained from Singapore Land Authority (SLA). Eight network buffers with the specified distance as 400 m, 800 m, 1000 m, 1200 m, 1600 m, 2000 m, 2400 m, 2800 m were created. UGS quantity at each buffer was then calculated for each respondent. In general, the size of network buffer with a specific distance is smaller than the circular buffer with the radius of the same distance. Both circular and network buffers are overlapping scales reflecting accumulative effects at smaller scales, while the nested buffers are completely independent and differentiate nearby and distant UGS.

### 2.3. Statistical Analyses

It was found that 23 respondents had very low quantity of vegetation cover and canopy cover. However, there exists greenery as shown in the Google map around these residences. To avoid wrong estimation, these respondents were excluded and thus the following analyses were conducted for the remaining 977 respondents. Exploratory data analysis was performed to gain a general understanding of the main characteristics of the respondents. Considering mental health is dichotomous, binary logistic regression models were used to calculate the odds ratios (ORs), standard errors and 95% confidence intervals (CIs) to show the associations of each UGS metric with mental health. All the statistical analyses were performed in IBM SPSS Statistics 24 (IBM Corp., Armonk, NY, USA).

Selecting confounders based on real survey data before the implementation of association estimation is crucial to rule out confounding effects. To determine the exact confounding factors that have influences on health outcomes, bivariate correlation analysis was first performed to examine the correlations between variables and the results were used as the primary diagnosis for multicollinearity. Afterwards, crosstabs analysis, chi-square test, and two sample T-test were employed to determine the potential confounding factors. These potential confounding factors were then examined as independent variables through binary logistic regression for mental health as dependent variable using forward stepwise selection method. Age and ethnicity have been reported to have significant effects on mental disorders for Singapore residents [55]. Therefore, in the following analyses, these two demographic factors were included as confounding factors. Multicollinearity has been checked by variance inflation factor (VIF) [56]. Finally, level of health problems, community involvement, housing ownership, BMI, gender, level of indoor physical activities, household income, smoking, residential status, children number, age, and ethnicity were included as finalized confounding variables in the regression models for adjustment. 

## 3. Results

### 3.1. Quantity of Different UGS Metrics at Different Scales

**Figure 3:**
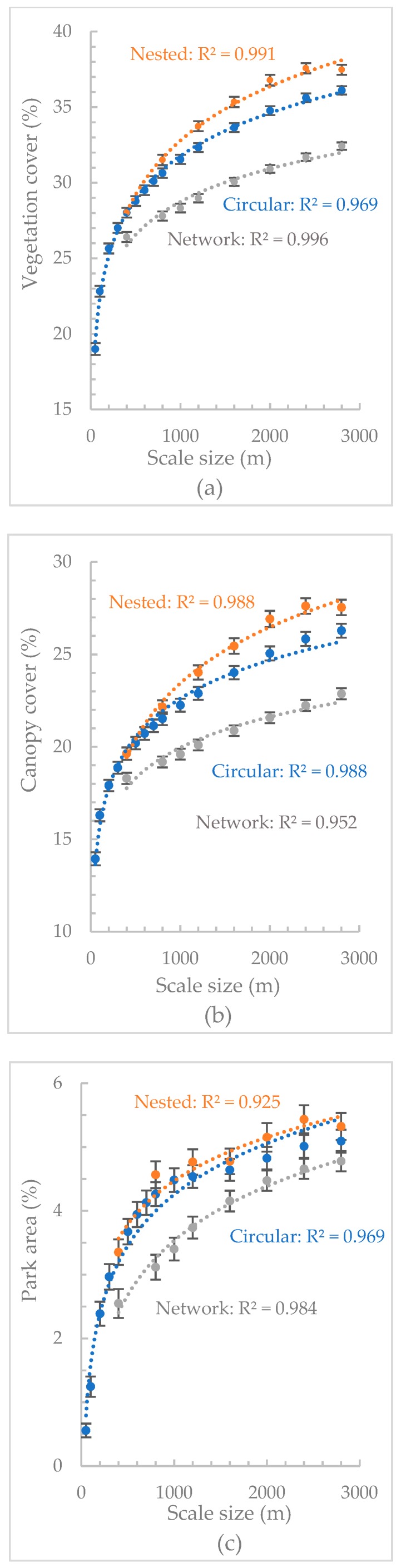
The average amount of (**a**) vegetation cover, (**b**) canopy cover, and (**c**) park area at different circular buffers (blue color), nested buffers (orange color), and network buffers (grey color).

In total, 44.08% of Singapore’s land was covered by vegetation cover and 35.77% of the land was covered by canopy cover. The total area of parks is 43.98 km^2^, which covers 5.98% of the whole island. Descriptive statistical analysis was conducted for the UGS quantity at each scale. As spatial scale increased, the average amount of vegetation cover, canopy cover and park area increased curvilinearly and the rate of increase declined (Figure 3). For all three UGS metrics, nested buffers consistently had higher quantity of UGS than circular and network buffers. 

### 3.2. Study Population

The respondents’ main characteristics are presented in Table 2. There were 12.2% of participants who reported poor mental health. Further information about the proportion of respondents having poor mental health within each subgroup is provided in Table 3. There was a higher proportion of females who had poor mental health compared to males, as well as among the middle-aged group and the elderly compared to the younger age groups. “Others” ethnicity also had a higher proportion of having poor mental health compared to the three main ethnic groups. There was a higher proportion of residents living in rental houses that had poor mental health compared to the residents living in owned houses. The respondents living in HDB (Public housing in Singapore managed by the Housing and Development Board) 1–2 room flat reported the highest proportion of having poor mental health, while the respondents living in private housing (landed property) reported the lowest proportions.

### 3.3. Comparison of Different UGS Metrics

**Figure 4:**
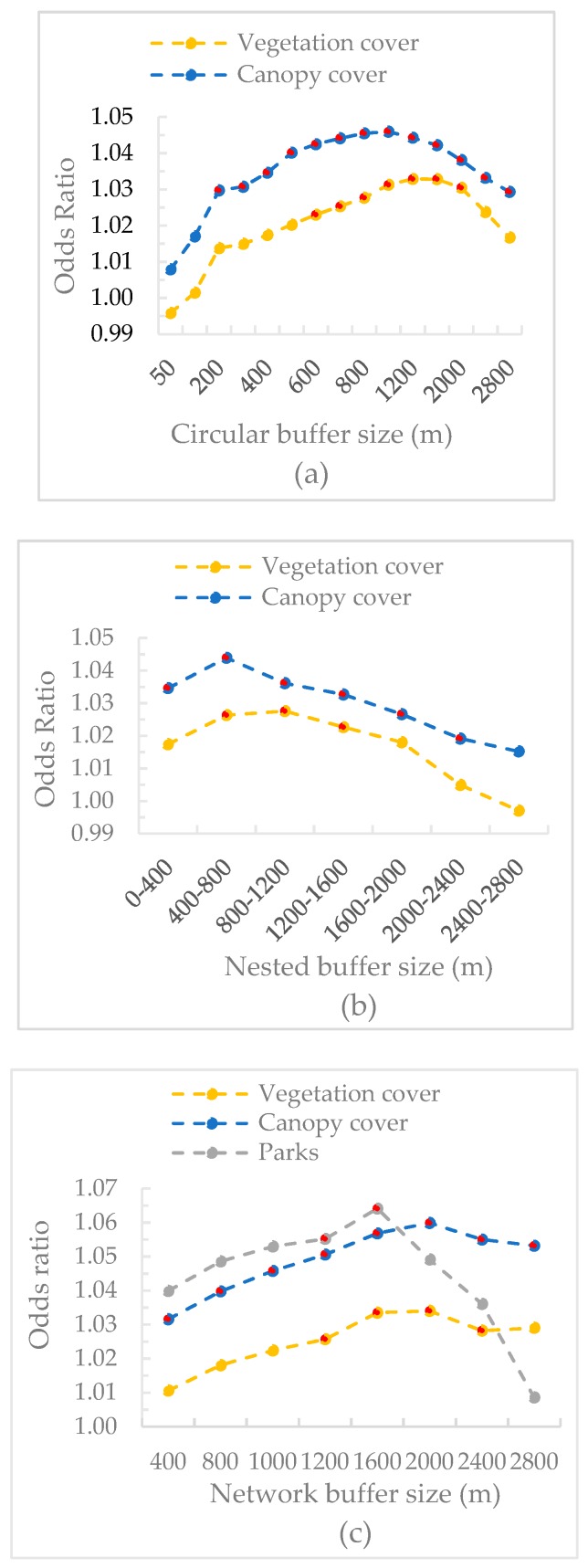
The associations between UGS quantity and mental health at circular buffers (**a**), nested buffers (**b**), and network buffers (**c**) (red dots are significant results at the significant level of 0.05).

The results of the associations between each UGS metric at each scale and mental health were provided in Supplementary Materials. The odds ratio of each UGS metric in different buffers can be seen in Figure 4. For both circular and nested buffers, except for park area, the other two UGS metrics were positively correlated with mental health. For network buffers, all the three UGS metrics had positive relationships with mental health and park area showed the highest association while vegetation cover showed the lowest association. The inverted-U curves in Figure 3 showed that the relationships between UGS quantity and mental health were only significant at certain scales. We interpreted these as the thresholds beyond and below which associations were significant. Vegetation cover at circular buffers from 600 m to 2000 m, nested buffers from 400–800 m to 1200–1600 m, and network buffers from 1200 m to 2400 m showed significant relationships. For canopy cover, circular buffers from 200 m to 2800 m, nested buffers from 0–400 m to 2000–2400 m, and network buffers from 400 m to 2800 m showed significant associations with mental health. Compared with vegetation cover, canopy cover had smaller minimum threshold scales and larger maximum threshold scales to manifest significant UGS-health relationships. Park area was only significantly related to mental health at network buffers between 1200 m and 1600 m. For all three types of buffers, the associations of canopy cover with mental health at all the scales were larger than that of vegetation cover.

### 3.4. Comparison of Different Spatial Scales

**Figure 5:**
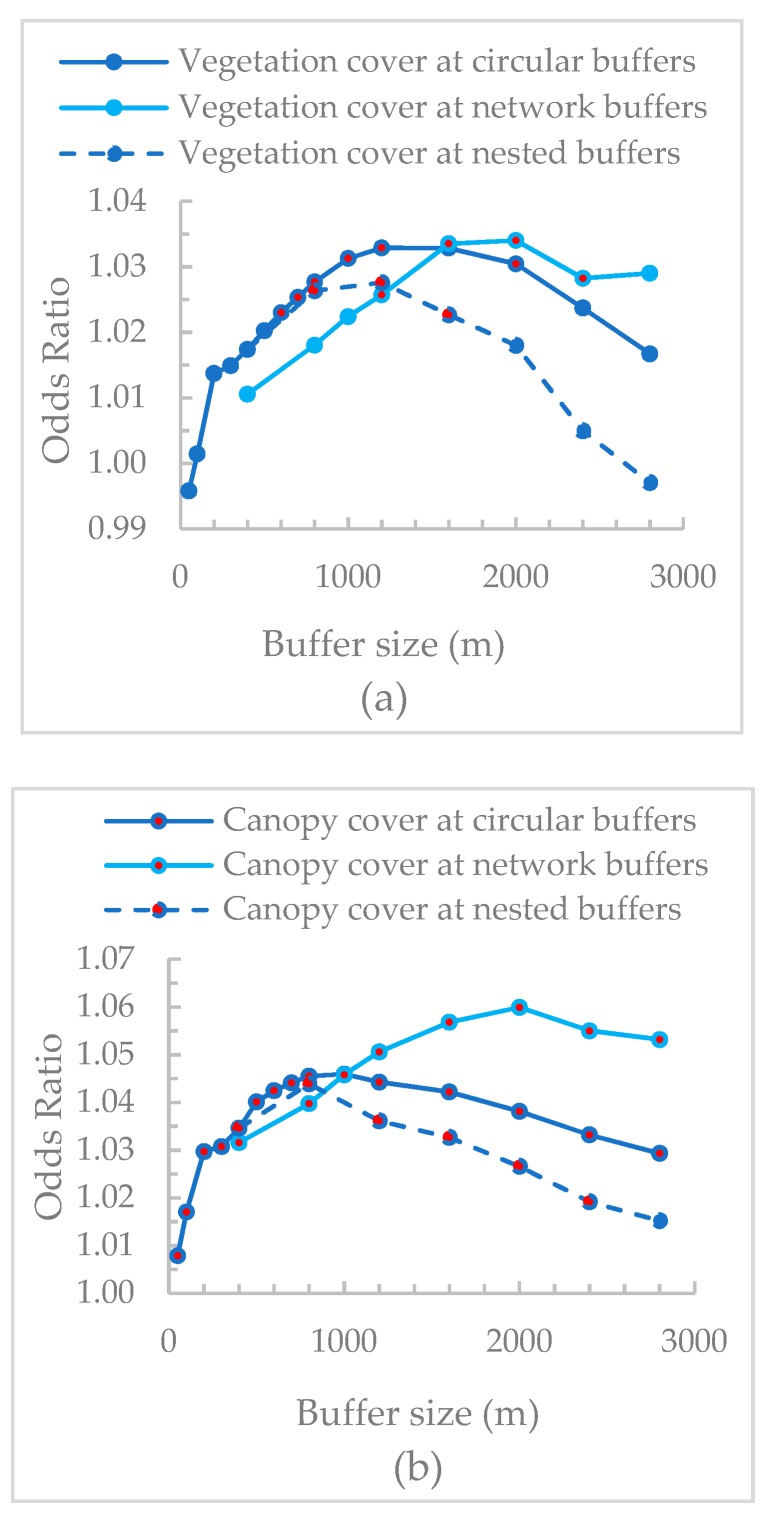

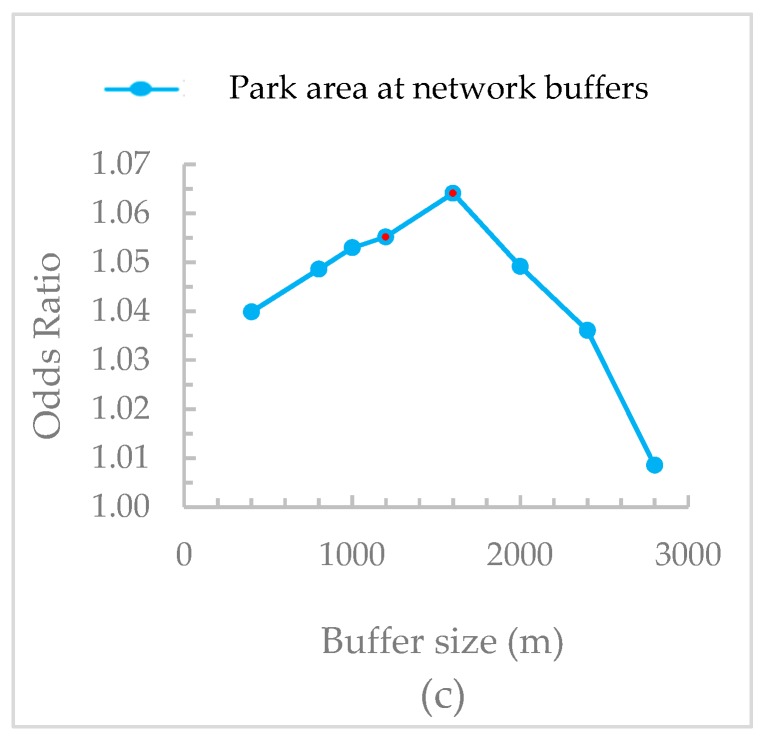
The associations of (**a**) vegetation cover, (**b**) canopy cover, and (**c**) park area with mental health at different buffers (red dots are significant results at the significant level of 0.05).

Figure 5 shows the impact of scale of analysis on odds ratio of each UGS metric. All the curves showed an initial increasing followed by decreasing trend. A similar pattern of the change trend of odds ratio at different types of buffers was observed for vegetation cover and canopy cover (Figure 5a,b). The associations with mental health at circular buffers were consistently higher than that at nested buffers (e.g., odds ratio at 800 m was higher than that at 400–800 m) and the difference between these two buffers increased with the increase of buffer size. Both vegetation cover at buffers smaller than 1600 m and canopy cover at buffers smaller than 1200 m showed higher associations at circular buffers compared to that at network buffers. Beyond these scales, the associations at network buffers were higher. Park area was unrelated to mental health across different circular buffers and nested buffers. At network buffers, park area showed a positive relationship with mental health but only at 1200 m and 1600 m (Figure 5c). 

## 4. Discussion

### 4.1. The Associations of Different UGS Metrics with Mental Health

All three types of measurements of UGS showed significant and positive relationships with mental health at certain spatial scales. In general, increasing vegetative cover, canopy cover and park area improved the odds ratio of having good mental health. However, the magnitude of UGS-mental health associations were different among different measures of green spaces. These findings confirmed that how UGS is measured has an impact on UGS-health associations, which highlights the importance of giving a clear description on the definition and measurement of UGS in this field [57]. Overall, the UGS-health relationship patterns for vegetation cover and canopy cover were similar, but that for park area was somewhat different. These findings suggest that there might be different mechanisms leading to mental health benefits obtained from land with different vegetation types. 

Park area at 1600 m network buffer showed the strongest association with mental health while vegetation cover showed the lowest association. This finding is in line with a study in Chicago showing that park spaces were more related to health and well-being than vegetation cover [30]. As suggested by researchers [1,50], health effects of UGS are mediated by different mechanisms. There are several possible reasons: parks have more well-designed vegetation and can generate a more restorative environment to relieve stress, as shown by several field studies [58,59]. Furthermore, as an environment more specifically designed for active usage, park can provide more opportunities for physical activities and social interactions [60]. These can trigger more health benefits. However, park area did not show the strongest associations across all scales. Except for two network buffers, park area was unrelated to mental health at other scales. This might be explained by the fact that residents tend to use the parks located at this range of distance from their residences. In the high-density residential estates in Singapore, neighborhood parks tend to be small and may not contain attractive features compared to regional parks, which are larger and have more amenities which are generally better used. At larger distances, the accessibility of parks decreased and they are less likely to be used on a frequent basis.

At most scales, canopy cover showed the strongest relationships with mental health. Vegetation cover also showed significant associations with mental health. The possible reason might be that visual contact with greenery and mitigation of environmental harms (e.g., air pollution and heat island effect) can provide more mental health benefits than physical activities and social interactions promoted by more structured greenery. As expected, canopy cover had stronger associations than vegetation cover with mental health. This agrees with the findings from a recent study in New York showing that trees have stronger and more consistent positive associations with self-reported health compared to grass [61]. Compared to grass, trees were reported to have stronger effects in improving the environment, such as reducing air pollution [62,63] and cooling urban areas [64], as well as providing more restorative benefits [65]. Therefore, vegetation cover which contains less trees reported the lowest associations with mental health. 

### 4.2. The Influence of Analytical Scale on UGS-Health Associations

The results showed that the strength and significance level of associations between UGS and health change with the spatial scale used for analysis. The results also confirmed Hypothesis 2 that there are certain thresholds beyond or below which UGS-health exhibit significant relationships. The minimum threshold scale for canopy cover was smaller than that for vegetation cover, while the minimum threshold scale for park area was larger than the other two UGS metrics. These findings form a better understanding of how the analytical scale can influence the associations between UGS and health.

In general, the circular buffers showed stronger relationships with mental health than nested buffers. This may be explained by the fact that nested buffers are the subset of circular buffers. For each UGS metric, network buffer observed the strongest relationship with mental health among the three types of buffers. Network buffers reflect the areas that can be reached by roads, and thus this finding suggests that the mechanism under UGS-health relationships may be related to the use of UGS. However, the hypothesis that network buffers will show stronger UGS-health relationships was not supported for all spatial scales studied. UGS at circular buffers showed higher associations than network buffers at buffers smaller than 1200 m or 1600 m, which are equivalent to the walking distance within 15 and 20 minutes. These findings might be explained by residents’ commuting behavior. In daily life, people may take shortcuts rather than roads to reach those nearby UGS located within 20 minutes. Conversely, to reach those UGS that are located farther away, use of roads via public or private transport is needed.

The UGS-health relationships at nested buffers indicated that it is not necessarily true that nearby greenery exhibits the greatest health benefits. Instead, mental health showed the strongest association with UGS at moderate buffer size from 400 m to 1600 m. This finding is consistent with many previous studies. A Dutch research team found the stress-buffering effects of green spaces on general health only at buffer with the radius of 3 km but not 1 km [46] and the effects of green spaces at 1–3 km nested buffer on both mental and general health were stronger than that at 1 km-radius buffer [44]. Another similar pattern was found in a Doetinchem cohort study, which reported significant associations of UGS with health at 1 km-radius buffer rather than 125 m-radius buffer [66]. In addition, the buffers between 400–1600 m in size are also similar with the results from two recent studies. A study conducted in Tokyo [45] examined the associations between NDVI at nested buffer of 0–100 m, 100–500 m, 500–1000 m, 1000–1500 m with mental health measured by GHQ-12 and found the strongest association at scale of 500–1000 m. A meta-analysis from another study also suggests that green spaces at scale of 500–999 m can best predict general health [38]. One possible reason for why the strongest associations were observed at medium scales could be that green spaces farther from residences tend to be more attractive than those nestled within residential areas [45]. The finding may also reflect the fact that the amount of canopy cover and park area are higher in distant than nearby spaces. The results obtained may also be due to green spaces that are farther away providing more opportunities for deeper restoration with a stronger sense of being away [46]. 

### 4.3. Study Limitations

This study has provided novel information but there are limitations in the study. First, although this cross-sectional study showed certain statistical associations at different buffers, it is still difficult to provide explanation of mechanisms leading to these outcomes, since correlation does not equal a causal relationship [67,68]. The second limitation is regarding the statistical analyses. Although the sample used in this study was a national representative sample, the sample size of 1000 is relatively small compared to a sample that is larger than 10,000 in many other studies [44,69,70,71]. The small sample may reduce the ability to detect small differences and the results may lack statistical power [46]. In addition, this study did not check for non-linearity. We suggest that other, more advanced statistical techniques (e.g., [72]) can be employed to strengthen the statistical power. The third limitation concerns the measurement of dependent and independent variables. This research applied questionnaire-based assessment to measure self-reported health. This, however, may provoke self-reporting response bias [73]. Moreover, this study only measured onefold aspect of UGS provision (UGS quantity at different distances). As our conceptual framework has suggested that UGS-health relationships are also influenced by UGS quality and people’s actual exposure to UGS [1], a greater understanding of these aspects is needed by incorporating the aspect of UGS quality explicitly in future studies. Fourth, why certain threshold levels were observed are not fully understood. Future studies should investigate possible mechanisms to improve our knowledge of causal mechanism and investigate possible reasons underlying threshold and scale-dependence.

### 4.4. Implications

Recent studies have provided indications that types of UGS and analytical scale influence outcomes of UGS-health studies, but to date, there have not been any systemic studies to examine the specificities of these two dependences [1,22,38,41,74,75]. To our best knowledge, this study is the most comprehensive study by far aiming to compare the role of how UGS are measured, and spatial scale of analysis to understand how methodological details may influence study results. More specifically, the study examined three types of UGS metrics, 15 circular buffers, seven nested buffers, and eight network buffers and their interactions on strength of health benefits. Through the results, we highlight that studies need to consider and provide explicit details on how UGS were measured and at which scale they were quantified to correlate with health responses.

UGS metrics which are better to capture the amount of vegetation or landscape features known for health-promotion effects are more useful in UGS-health studies. We suggest that in general, canopy cover is a better metric than vegetation cover for UGS-health studies. The practical implication is that just concentrating on increasing total vegetation alone is likely to be too blunt a strategy to promote health benefits from green space exposure. The important elements of vegetation for health promotion or mental restoration, in particular, trees and green open spaces with strong recreational value, appear to be more important as suggested through our study. There is also scope to explore specific landscape features and the way landscapes are designed to achieve better mental restoration [76]. More work in this aspect should be conducted for UGS.

As our review highlighted, there are a limited number of studies conducted in tropical areas [1]. This study strengthened the evidence of positive health effects of greenery for Singapore as a tropical, compact and green city. This is important, as there was only one study which investigated the UGS-well-being relationship in Singapore, and which had concluded that green spaces did not foster well-being [77]. In contrast to the sample of university students applied in Saw et al.’s study, the present study used a national representative sample and observed positive and significant associations between UGS and health. It helps to add weight to the argument that UGS is not just for aesthetic improvement to urban landscapes, ecological or economic benefits, but needs to be considered as an important health intervention measure as part of the suite of health promotion activities already undertaken by the state. The results will thus be an important part of evidence-based policy making locally for operationalizing the concept of fostering green space exposure as an upstream health intervention measure.

## 5. Conclusions

This study achieved its aim of examining how the choice of UGS measure and analytical scale can impact UGS-health relationships in Singapore. Overall, canopy cover showed the strongest associations with mental health at most scales. The strongest UGS-health relationships were found at medium scales. We suggest that future studies should carefully choose the measures for UGS and carefully determine the boundary to measure UGS. In fact, analyzing UGS-health associations at multiple scales within a study is also recommended.

## Figures and Tables

**Table 1:** Sampling stratification by demographic characteristics and housing type.

Characteristics	Stratification	Target	Collected	Difference
Gender	Male	49.1%	45%	−4.1%
	Female	50.9%	55%	+4.1%
Ethnicity	Chinese	74.3%	72.2%	−2.1%
	Malays	13.4%	15.3%	+1.9%
	Indians	9.1%	9.3%	+0.2%
	Others	3.2%	3.2%	0
Housing type	Total HDB Dwellings ^1^	81.1%	81.1%	0
	Condominiums, Other Apartments	12.5%	12.6%	+0.1%
	Landed Properties	6.4%	6.3%	−0.1%

^1^ Public housing in Singapore managed by the Housing and Development Board (HDB).

**Table 2:** Sampling stratification by demographic characteristics and housing type.

Sociodemographic	Total (*N* = 977) *n* (% of Total Sample)
**Gender**	
Men	442 (45.2%)
Women	535 (54.8%)
**Age**	
21–24	49 (5.0%)
25–34	123 (12.6%)
35–44	190 (19.4%)
45–54	183 (18.7%)
55–64	194 (19.9%)
65–74	157 (16.1%)
75–84	72 (7.4%)
85 & over	9 (0.9%)
**Ethnicity**	
Chinese	702 (71.9%)
Malay	152 (15.6%)
Indian	91 (9.3%)
Others	32 (3.3%)
**House ownership**	
Owned	896 (91.7%)
Rental	81 (8.3%)
**Housing type**	
HDB1 1–2 room flat	61 (6.2%)
HDB 3 room flat	187 (19.1%)
HDB 4 room flat	325 (33.3%)
HDB 5 room or executive flat	218 (22.3%)
Private condominium/apartment	123 (12.6%)
Private housing (landed property)	63 (6.4%)
**Marital status**	
Single	185 (18.9%)
Married	701 (71.8%)
Widowed	51 (5.2%)
Divorced/Separated	40 (4.1%)
**Employment status**	
Full-time	376 (38.5%)
Part-time	106 (10.8%)
Self-employed	65 (6.7%)
Retired	168 (17.2%)
Student/NSF2	25 (2.6%)
Homemaker/Unemployed	237 (24.3%)
**Highest Education level**	
Below Secondary	224 (22.9%)
Secondary	280 (28.7%)
Post-Secondary (Non-Tertiary)	87 (8.9%)
Diploma & Professional Qualification	170 (17.4%)
University & above	216 (22.1%)

HDB: Public housing in Singapore managed by the Housing and Development Board; NSF: National Servicemen Full-time.

**Table 3:** Percentage of respondents with poor mental health.

Sociodemographic	Percentage of Respondents with Poor Mental Health
**Gender**	
Men	10.0%
Women	14.0%
**Age**	
21–24	8.2%
25–34	12.2%
35–44	7.9%
45–54	14.2%
55–64	13.4%
65–74	14.7%
75–84	13.9%
85 & over	0%
**Ethnicity**	
Chinese	12.4%
Malay	9.2%
Indian	11.0%
Others	25.00%
**House ownership**	
Owned	11.3%
Rental	22.2%
**Housing type**	
HDB 1–2 room flat	29.5%
HDB 3 room flat	11.2%
HDB 4 room flat	11.7%
HDB 5 room or executive flat	11.9%
Private condominium/apartment	9.8%
Private housing (landed property)	6.4%

## Supplementary Materials

The following are available online at http://www.mdpi.com/1660-4601/16/4/578/s1, Associations between urban green spaces and health are dependent on the analytical scale and how urban green spaces are measured.
