# Supplementary material for: Associations between Urban Green Spaces and Health are Dependent on the Analytical Scale and How Urban Green Spaces are Measured
Source: Int J Environ Res Public Health. 2019 Feb 16;16(4):578. doi: 10.3390/ijerph16040578 (PMC6406785; doi:10.3390/ijerph16040578)
Supplement: Supplementary file 1 [file ijerph-16-00578-s001.pdf]

Supplementary Materials for paper

Associations between urban green spaces and health are dependent on the analytical scale and how urban green spaces are measured

Associations between vegetation cover and mental health

For circular buffers, the associations of the amount of vegetation cover with mental health was shown only at scales from 600 m to 2000 m (Table S1). The strongest association with mental health (OR=1.033, 95% CI: 1.009-1.059, p<0.01) was found at 1200 m and 1600 m. For nested buffers, only vegetation cover at buffers with radius ranging from 400 m to 1600 m were significantly associated with mental health and the strongest association (OR=1.028, 95% CI: 1.006-1.051, p<0.05) was found at 800-1200 m. For vegetation cover at network buffers, the significant associations were found at the scales ranging from 1200 m to 2400 m. The strongest association was found at 1600 m (OR=1.034, 95% CI: 1.007-1.062, p<0.05) and 2000 m (OR=1.034, 95% CI: 1.006-1.064, p<0.05).

Table S1. Binary logistic regression for mental health and vegetation cover at different buffers

Type of spatial scale	Buffer size (m), fully adjusted[1]	Odds Ratio	95% C.I. for Odds Ratio		p-value
			Lower	Upper	
Circular buffers	50	0.996	0.980	1.012	0.605
	100	1.001	0.984	1.020	0.872
	200	1.014	0.993	1.035	0.193
	300	1.015	0.994	1.037	0.165
	400	1.017	0.997	1.039	0.107
	500	1.020	0.999	1.043	0.066
	600	1.023*	1.002	1.046	0.039
	700	1.025*	1.004	1.049	0.025
	800	1.028*	1.006	1.051	0.016
	1000	1.031**	1.008	1.056	0.009
	1200	1.033**	1.009	1.059	0.008
	1600	1.033**	1.009	1.059	0.009
	2000	1.030*	1.006	1.056	0.015
	2400	1.024	1.000	1.049	0.056
	2800	1.017	0.993	1.042	0.178
Nested buffers	0-400	1.017	0.997	1.039	0.107
	400-800	1.026*	1.006	1.048	0.013
	800-1200	1.028*	1.006	1.051	0.014
	1200-1600	1.023*	1.002	1.044	0.032
	1600-2000	1.018	0.999	1.039	0.074
	2000-2400	1.005	0.985	1.025	0.622
	2400-2800	0.997	0.978	1.017	0.772
Network buffers	400	1.011	0.990	1.032	0.317
	800	1.018	0.995	1.042	0.128

	1000	1.022	0.999	1.048	0.067
	1200	1.026*	1.002	1.052	0.041
	1600	1.034*	1.007	1.062	0.015
	2000	1.034*	1.006	1.064	0.018
	2400	1.028*	1.001	1.058	0.050
	2800	1.029	1.001	1.060	0.051
1. Adjusted for level of health problems, community involvement, housing ownership, BMI, gender, level of indoor physical activities, household income, smoking, residential status, children number, age, ethnicity<br>***. p≤ 0.001; **. p≤ 0.01; *. p≤ 0.05					

Association between canopy cover and mental health

The associations between canopy cover and mental health were significant at all the buffers except for the circular buffers of 50 m, 100 m, and nested buffer of 2400-2800 m (Table S2). The strongest association for circular buffers (OR=1.046, 95% CI: 1.022-1.072, p<0.001) was found at 1000 m. For nested buffers, the strongest relationship (OR=1.044, 95% CI: 1.021-1.069, p<0.001) was found at 400-800 m. All the network buffers showed significant associations between canopy cover and mental health and the strongest association (OR=1.060, 95% CI: 1.032-1.091, p<0.001) was found at 2000 m.

Table S2. Binary logistic regression for mental health and canopy cover at different buffers

Type of spatial scale	Buffer size (m), fully adjusted[1]	Odds Ratio	95% C.I. for Odds Ratio		p-value
			Lower	Upper	
Circular buffers	50	1.008	0.990	1.027	0.398
	100	1.017	0.997	1.039	0.109
	200	1.030*	1.007	1.055	0.014
	300	1.031**	1.008	1.056	0.010
	400	1.035**	1.012	1.060	0.004
	500	1.040***	1.017	1.066	0.001
	600	1.042***	1.019	1.068	0.001
	700	1.044***	1.020	1.070	0.000
	800	1.045***	1.022	1.072	0.000
	1000	1.046***	1.022	1.072	0.000
	1200	1.044***	1.021	1.070	0.000
	1600	1.042***	1.020	1.067	0.000
	2000	1.038***	1.017	1.061	0.001
	2400	1.033**	1.013	1.055	0.002
	2800	1.029**	1.009	1.051	0.005
Nested buffers	0-400	1.035**	1.012	1.060	0.004
	400-800	1.044***	1.021	1.069	0.000
	800-1200	1.036***	1.016	1.058	0.001
	1200-1600	1.033***	1.014	1.054	0.001

	1600-2000	1.027**	1.009	1.046	0.004
	2000-2400	1.019*	1.002	1.038	0.034
	2400-2800	1.015	0.998	1.034	0.092
Network buffers	400	1.032*	1.007	1.058	0.012
	800	1.040**	1.014	1.068	0.003
	1000	1.046***	1.019	1.075	0.001
	1200	1.051***	1.024	1.080	0.000
	1600	1.057***	1.029	1.087	0.000
	2000	1.060***	1.032	1.091	0.000
	2400	1.055***	1.028	1.085	0.000
	2800	1.053***	1.026	1.083	0.000
1. Adjusted for level of health problems, community involvement, housing ownership, BMI, gender, level of indoor physical activities, household income, smoking, residential status, children number, age, ethnicity<br>***. p≤ 0.001; **. p≤ 0.01; *. p≤ 0.05					

Association between park area and mental health

As shown in Table S3, park area at all the circular and nested buffers were not correlated with mental health. Parks at network buffers with the distance of 1200 m and 1600 m were significantly associated with mental health. The strongest association (OR=1.064, 95% CI: 1.008-1.124, $p<0.05$) was observed at 1600 m.

Table S3. Binary logistic regression for mental health and park area at different buffers

Type of spatial scale	Buffer size (m), fully adjusted[1]	Odds Ratio	95% C.I. for Odds Ratio		p-value
			Lower	Upper	
Circular buffers	50	1.057	0.975	1.211	0.286
	100	1.017	0.972	1.081	0.535
	200	1.030	0.987	1.088	0.229
	300	1.030	0.990	1.081	0.184
	400	1.041	1.000	1.093	0.072
	500	1.041	1.001	1.090	0.062
	600	1.039	1.000	1.087	0.068
	700	1.040	1.000	1.087	0.066
	800	1.042	1.001	1.091	0.058
	1000	1.043	1.001	1.094	0.061
	1200	1.044	1.000	1.097	0.067
	1600	1.031	0.988	1.084	0.191
	2000	1.024	0.984	1.074	0.283
	2400	1.021	0.982	1.069	0.327
	2800	1.018	0.980	1.065	0.385

Nested buffers	0-400	1.041	1.000	1.093	0.072
	400-800	1.034	0.998	1.077	0.086
	800-1200	1.030	0.993	1.075	0.142
	1200-1600	1.008	0.976	1.047	0.647
	1600-2000	1.009	0.980	1.044	0.561
	2000-2400	1.011	0.981	1.047	0.507
	2400-2800	1.008	0.977	1.045	0.627
Network buffers	400	1.040	0.987	1.095	0.141
	800	1.049	0.992	1.108	0.093
	1000	1.053	0.997	1.112	0.063
	1200	1.055*	1.001	1.112	0.046
	1600	1.064*	1.008	1.124	0.025
	2000	1.049	0.995	1.106	0.076
	2400	1.036	0.983	1.092	0.189
	2800	1.009	0.962	1.057	0.724

1. Adjusted for level of health problems, community involvement, housing ownership, BMI, gender, level of indoor physical activities, household income, smoking, residential status, children number, age, ethnicity

*. $p \leq 0.05$
